# Reactive Sulfur Species (RSS) in Physiological and Pathological Conditions and in Therapy

**DOI:** 10.3390/antiox11081576

**Published:** 2022-08-15

**Authors:** Anna Bilska-Wilkosz, Małgorzata Iciek

**Affiliations:** Faculty of Medicine, Jagiellonian University Medical College, 31-008 Kraków, Poland

Sulfur is a multivalent and nonmetallic chemical element with the symbol S and the atomic number 16. The abundance of sulfur in the earth’s crust is around 0.03%, and it is an essential component of all living cells. Plants contain, on average, approximately 1 g of sulfur/kg dry weight. The average human body (70 kg) contains about 140 g of sulfur, especially thiol compounds, which play a key role in metabolism. The most important biologically-active thiol compounds include cysteine (Cys), homocysteine (Hcy), a reduced form of lipoic acid (LA), i.e., dihydrolipoic acid (DHLA), reduced glutathione (GSH) and coenzyme A (CoA). It should also be mentioned that the sulfur-containing amino acid, methionine (Met), is one of the essential amino acids for the human body. Met can be converted to S-adenosylmethionine (SAM), the major methyl group donor in one-carbon metabolism. After the donation of the methyl group, S-adenosylhomocysteine (SAH) is formed, which is hydrolyzed to Hcy. There are two major disposal pathways for Hcy: the remethylation process, which is the conversion to Met, and the transsulfuration process, resulting in the formation of Cys—the sulfur of Met becomes the sulfur of Cys.

Cys is the main source of sulfur in the animal and human body. The sulfur of Cys is used in the synthesis of thiol compounds, including lipoic acid (LA), glutathione (GSH), and coenzyme A (CoA). Thiol compounds can exist in tissues in a free form and be bound to proteins in a reduced and oxidized form, and all of these forms compose a dynamic system known as “thiol redox status”.

In the body, Cys is metabolized via two pathways. The first one is an aerobic pathway, which is a series of processes leading to taurine and inorganic sulfate. The second route is an anaerobic pathway, leading to the formation of reactive sulfur species (RSS). Representative RSS include hydrogen sulfide (H_2_S), hydrogen persulfide (H-S-S-H), organic persulfides (R-S-S-H), and polysulfides (HSS_n_H, RSS_n_H, RSS_n_R, *n* ≥ 1). Per- and polysulfides are H_2_S oxidation products that contain one or more zero-valent sulfur atoms, called sulfane sulfur. Sulfane sulfur-containing compounds also include thiosulfates (S=SO_3_^2−^) and elemental sulfur (S_8_).

The greatest fascination with the metabolism and role of sulfur compounds existed in the 1960s and 1970s. In the next two decades, the marginalization of this topic was observed, and then, at the beginning of the new millennium, this subject was revived again. This rapid renewed interest in the physiological role of sulfur was initiated in 1996 by the studies of Abe and Kimura [1], who showed that H_2_S was synthesized in the hippocampus via an enzyme-dependent pathway and suggested that endogenous H_2_S functioned as a neuromodulator in the brain.

Thus, the launch of this Special Issue in 2021 coincided with the 25th anniversary of the comeback of the topics related to the biochemistry and pharmacology of sulfur compounds to science. This Special Issue is entitled ‘Reactive Sulfur Species (RSS) in Physiological and Pathological Conditions and in Therapy’ and contains nine papers, five of which are original papers, and four are reviews.

An original paper by Pharoah et al. addresses the cardioprotective role of various RSS precursors [2]. This study was performed on the Langendorff ex vivo myocardial ischemia/reperfusion (I/R) injury model. The results obtained by the authors showed that all of the tested RSS were capable of reducing the extent of irreversible damage to the heart, with one of the precursors of RSSH being the most effective. The compounds releasing RSSH are the alkylamine-substituted perthiocarbamates that efficiently stimulate RSSH release with half-lives ranging from 1.4 to 484 min in the presence of β-4-hydroxyphenyl) ethyl iodoacetamide (HPE-IAM) as an RSSH trap [3]. Furthermore, using the H9c2 embryonic rat heart myoblasts cell line, the authors showed that one of the precursors of RSSH with a half-life of 16.7 min significantly decreased cellular mitochondrial respiration. Furthermore, the authors observed that the lowered metabolic demand of H9c2 cardiomyocytes in the early phase of reperfusion correlated with the increased viability of these cells following the hypoxic episode. A research paper by Skibska et al. [4] also focused on the cardiovascular system. The aim of this study was to evaluate the effects of LA on oxidative stress parameters and inflammation in the ventricles and atria of the heart in rats under lipopolysaccharide (Escherichia coli LPS 026:B6)-induced oxidative stress. LA, and its reduced form DHLA, are considered to be powerful antioxidants. Therapeutically, LA is used in a variety of diseases, including cardiovascular disorders. Dudek et al. indicated that LA protected the heart against myocardial post-I/R arrhythmias via potassium ATP-sensitive channels in isolated rat hearts [5]. The research by Sokołowska et al. has proven a beneficial effect of LA on cyanate toxicity in the rat heart [6]. However, in the aforementioned paper by Skibska et al., the authors showed that the administration of LPS to rats resulted in the inflammation of the ventricles and atria and oxidative stress, confirmed by an increase in thiobarbituric-acid-reactive substance (TBARS), hydrogen peroxide (H_2_O_2_), tumor necrosis factor (TNF-α), and pro-inflammatory cytokine interleukin-6 (IL-6). In addition, it has been shown that in the ventricles and atria, the level of total sulfhydryl groups and GSH and the activity of superoxide dismutase (SOD) decreased. In contrast, in the group of animals that received LA after the injection of LPS, the level of TNF-α, IL-6, TBARS, and H_2_O_2_ in the atria and ventricles significantly decreased, and the activity of SOD and the level of total sulfhydryl groups significantly increased. This indicates the protective effects of LA administration against LPS-induced oxidative stress in the rat heart. It is worth mentioning that currently, an increasing body of literature data indicate the possibility of also using LA in the treatment or prevention of COVID-19 [7]. In this Special Issue, there is one review paper on the pharmacological activity of various endogenous or exogenous reactive sulfur compounds in diseases caused by SARS-CoV-2 [8]. This article reviews the current knowledge about H_2_S, N-acetylcysteine (NAC), GSH, and the already mentioned LA, and discusses the possibility and the results of their use in the treatment or prophylaxis of COVID-19. In this context, the authors also discussed other less-known sulfur compounds, such as erdosteine and ergothioneine (ET). A large part of the article is also dedicated to disulfiram (DSF), which is a well-known drug used to support the treatment of alcoholism. DSF, which is structurally a disulfide, is rarely discussed in papers dealing with the biological properties of sulfur compounds, perhaps because it is not produced by living organisms but is a synthetic drug. The main active metabolite of DSF, formed in vivo, N,N-diethyldithiocarbamate (DDC), structurally is a thiol compound. Today, DSF belongs to the “fashionable” group of “old drugs with a new face”. DSF today remains a marginal molecule in the treatment of alcoholism, but increasingly more often it is indicated as a potential anticancer, antibacterial, and antiviral drug. In the case of the SARS-CoV-2 virus, it has been documented that two cysteine proteases play a pivotal role in mediating the replication and transcription of this virus. It is the main protease (Mpro) and the papain-like protease (PLpro). In the review cited above, the authors discuss a possible mechanism of modification of the catalytic Cys145 of SARS-CoV-2 Mpro by DSF and its metabolites, leading to the inhibition of Mpro activity. An interesting look at sulfur compounds is presented in another review paper by Tiganescu and colleagues [9]. The authors note that sulfur compounds are generally divided into two groups. The first one, called Organic Sulfur Compounds (OSCs), is often addressed in a more chemical context. The second group consists of the already-mentioned RSS and is more often defined in a more biological context. In both these groups, however, there are also the so-called “Volatile Sulfur Compounds (VSCs) which stand out as they tend to combine high reactivity and hence biological activity with a unique air-bound bioavailability”. In their article, the authors presented the structure and biological activity of many VSCs, starting with the simple volatile inorganic sulfur compounds, such as H_2_S and sulfur dioxide (SO_2_), through the active ingredients contained in commonly-known plants, such as garlic, onion, mustard, and broccoli, and finishing with many lesser-known sulfur compounds appearing in shiitake mushroom and in durian fruit. It is worth noting that SO_2_—similar to H_2_S—is a double-faced molecule. Until recently, SO_2_ was only thought to be a toxic gas and an air pollutant, the molecule responsible for the occurrence of acid rain, and was generally viewed as a foe. The only good side of SO_2_ noted was that it could be used to fumigate hospital wards and as a preservative for nuts, fruits, and wine. Contemporary research focuses, on the other hand, on the beneficial effects of SO_2_ on living organisms. It is known today that SO_2_ and its derivatives are present in mammalian organisms in micromolar concentrations. It has also been shown in the in vivo studies that this molecule reduces myocardial I/R injury and atherosclerotic lesions. To wrap up the topic of VSCs, there are some additional words about the durian fruit that is cultivated in Southeast Asia countries, such as Malaysia, Thailand, Indonesia, and the Philippines. It is considered the most smelly fruit in the world. The unique aroma of durian fruit is associated, inter alia, with the presence of large amounts of VSCs, such as tiols, sulfides, polisulfides, thioacetals, thioesters, and thiolanes. The studies conducted on various experimental models, both in vitro and in vivo, have proven that durian possesses anti-atherosclerotic, anti-hyperglycemic, probiotic, and anti-proliferative effects. Moreover, durian is used as an ingredient in some foods. Despite its very bad smell, durian is said to be very tasty (sic!). Due to its smell, the durian fruit is banned from eating in most public places in South Asia. For “beginner” tourists from other parts of the world, it may seem funny when, for example, while traveling by train, to see a sign with the words “No durians”. There is now scientific evidence of the adverse, sometimes even lethal, effect of ingesting durian while imbibing alcohol. Symptoms are reminiscent of the DSF–ethanol reaction (DER) arising from the inhibition of aldehyde dehydrogenase (ALDH). Sulfur compounds present in the durian fruit are responsible for the inhibition of ALDH activity [10]. The inhibitory effects of sulfur-containing compounds from natural products on ALDH have also been described by our group [11].

Pophal et al.—the authors of the next paper—propose a new method for the determination of fractional exhaled nitric oxide (FENO) in the breath of humans [12]. Nitric oxide (NO) is considered to be a measure of inflammation in the respiratory system. Currently, it is already known that the concentration of exhaled NO is markedly elevated in bronchial asthma and is positively related to the degree of inflammation in eosinophilic disorders. The biological activity of NO is determined not only by its biosynthesis but also by its transport and storage capabilities, as well as the speed and direction of biodegradation. Thus, the physiological, therapeutic, as well as pathological effects of NO, depend on the presence of its various redox forms known as reactive nitrogen species (RNS). One of the properties of RNS is related to its ability to react with compounds that have a sulfhydryl group. As a result of this process, the so-called S-nitrosothiols (R-S-NO, SNT) are formed. The history of the discovery of SNT dates back to the 1950s when Castellani and Niven indicated that in order to inhibit the growth of bacteria by sodium nitrite, the presence of compounds with a thiol group was necessary [13]. However, the greatest interest in the interaction between NO and thiols was sparked by the pioneering research by Stamler, who, for the first time, documented the formation of SNT in the body [14]. One of the endogenous SNT, namely S-nitrosoglutathione (GSNO), plays a critical role in NO signaling and is a source of bioavailable NO. Pophal et al. hypothesized that FENO could reflect airway R-S-NO concentrations. To test this hypothesis, they first studied the relationship between FENO and airway R-S-NO in patients endotracheally intubated for respiratory failure. The results obtained by the authors indicate that FENO is indicative of, at least in part, GSNO breakdown. In the paper, it was shown that, unlike GSNO, NO is not present in the lung in physiologically relevant concentrations. At the end of their article, the authors wrote: “These studies demonstrate that the presence and metabolism of GSNO affects FENO. This relationship between GSNO and NO has important implications. FENO levels are not always useful as diagnostic tests for airway inflammation but could be employed as readouts in challenge tests in the lung function lab that are designed to evaluate airway pH, airway microbiome, and GSNO metabolic status. GSNO metabolism, in turn, is increasingly recognized as important to asthma pathophysiology” [12]. This paper is very interesting.

RSS play an essential role in the metabolic processes of all living organisms, so their determination in biological samples is known to be crucial for a better understanding of their function. However, the determination of RSS in biological samples is difficult and is a fundamental challenge for researchers. Thus, the readers may be interested in the original article by Roda et al., who reported the optimization of the analytical method for RSS detection in biological samples based on the analysis of the monobromobimane (MBB) derivative of sulfide using high-performance liquid chromatography with fluorescence detection (HPLC-FLD) [15]. The method itself is one of the popular derivatization protocols used for RSS detection; however, it is not free of limitations and some erratic results. Therefore, an improvement of the protocol aimed to make this method more reliable and useful for the analysis of biological samples is justified. The authors reported the optimization of the temperature, MBB concentration, retention time, and sample-handling. Using the proposed improvements, the authors demonstrated the precision and reliability of this modified method by determining various forms of RSS in the plasma of patients before and after the inhalation of vapors rich in H_2_S. The authors showed that their methodology is sufficiently sensitive and specific to detect an increase in RSS species and allows a selective evaluation of the different RSS pools in human serum. This paper has elements of novelty and innovations.

The next original article by Wang et al. refers to rhodanese 2 (RDL2) from the *Saccharomyces cerevisiae* (*S. cerevisiae*) [16]. Rhodaneses (thiosulfate:cyanide sulfotransferases; EC 2.8.1.1) were first discovered in mammalian tissues. Until recently, it was thought that the main biological function of rhodaneses involved the transformation of toxic cyanide to much less-toxic thiocyanate (rhodanate) with the use of an outer sulfur atom of thiosulfate. Now it is known that rhodaneses are present in all three kingdoms of life and are involved in various biological processes. *S. cerevisiae* grows with both sulfate and thiosulfate as a source of sulfur. It is known that yeast needs less energy to reduce thiosulfate than sulfate, and maybe for this reason, they produce more ethanol when using thiosulfate than using sulfate [17]. It is also known that there are two rhodaneses in yeast cells, Rdl1 and Rdl2, that can convert thiosulfate to a persulfide and sulfite. The persulfide is reduced by cellular thiols to H_2_S, and sulfite is reduced by sulfite reductase to H_2_S. The *S. cerevisiae* RDL1 is critical for thiosulfate assimilation through converting thiosulfate to glutathione persulfide (GSSH).

Rdl1 transfers the sulfane sulfur from thiosulfate also to other thiols, such as coenzyme A, Cys, and dithiothreitol (DTT), to form persulfides. It should be added, however, that the physiological concentrations of GSH are very high in yeast cells, up to 10 mM, and, therefore, it is the main sulfane sulfur acceptor in the Rdl1 catalyzed reactions. It has been discovered that Rdl2 is the main enzyme responsible for RSS generation in *S. cerevisiae* mitochondria, in which no sulfide:quinone oxidoreductase is present. Rdl2 releases sulfane sulfur atoms from stable sulfane sulfur carriers, such as thiosulfate and RS_n_R, to produce RSS [18]. In the paper published in this Special Issue, the authors investigated *S. cerevisiae*-derived RDL2, its 3D structure, and its role in the decomposition of thiosulfate. They have found that in the RDL2 catalytic site, the cysteine persulfide (Cys-SSH) is formed and that arginine (Arg) residue of the enzyme active-site loop plays an important role in the mechanism of thiosulfate decomposition. On the basis of their results (LC-MS/MS analysis, HPLC, and fluorimetric analysis), the authors propose a model to explain the mechanism by which rhodanese subtracts sulfane sulfur from stable thiosulfate to form an unstable persulfide. It is interesting and valuable information concerning the generation of RSS.

Furthermore, glycosaminoglycans (GAGs), called mucopolysaccharides, are important sulfur compounds in the body. GAGs are degraded in the lysosomes. The impaired degradation of GAGs is the cause of several diseases collectively known as mucopolysaccharidoses (MPSs). The problem with MPS III, called Sanfilippo syndrome type B, is the subject of the review paper by Kaczor-Kamińska et al. [19]. This disorder results in the massive lysosomal storage of heparan sulfate (HS) due to a deficit or complete lack of activity of alpha-N-acetylglucosaminidase (EC 3.2.1.50), caused by a mutation in the N-alpha-acetylglucosaminidase (NAGLU) gene. Generally, MPSs are inherited in an autosomal recessive mode. The exception is MPS type II (Hunter’s disease), the inheritance of which is linked to the X chromosome. In general, GAGs take the form of a long, unbranched heteropolysaccharide chain composed of a repeating disaccharide unit, in which amino sugar is always one of the components (hence the name GAGs), i.e., D-glucosamine or D-galactosamine, in which the amino group is usually acetylated. The amino sugar may also be sulfated on carbon 4 or 6 or on nonacetylated nitrogen in a sulfotransferase-catalyzed process in the presence of 3′-phosphoadenosine-5′-phosphosulfate (PAPS), which serves as a universal sulfate donor compound for all sulfotransferase reactions [20]. In mammals, sulfate for the synthesis of PAPS is derived from the aerobic metabolism of Cys. The authors, based on their previous studies [21,22], underline the fact that HS accumulation affects anaerobic Cys metabolism leading to sulfane sulfur-containing compounds. It is worth pointing out that Sanfilippo syndrome is characterized by severe central nervous system (CNS) degeneration with mild somatic symptoms. The main involvement of the CNS is unique to MPS III. It is necessary to remember that children with Sanfilippo syndrome are born asymptomatic, usually appear completely healthy, and most often develop normally until the second year of life. The symptoms, namely a marked hyperactivity, aggressive behavior, sleep disturbances, and mental retardation, occur only between 2 and 6 years of age. Unfortunately, the disease is incurable, and usually, the affected individuals die by the age of twenty. Therefore, we share the authors’ hope that a better diagnosis of the interrelationships between aerobic and anaerobic Cys metabolism in patients with Sanfilippo syndrome will allow for the development of a new curative strategy, enabling effective therapy for people suffering from this disease.

Finally, we would recommend a review paper by Moosmann and Hajieva entitled ‘Probing the Role of Cysteine Thiyl Radicals in Biology: Eminently Dangerous, Difficult to Scavenge’ [23]. Protein thiyl radicals (RS^•^) are RSS-generated in the redox processes of thiols and disulfides. They participate in many important biochemical processes in the body. On the other hand, thiyl radicals are dangerous to the body due to their high reactivity that can damage important cell components, mainly membrane proteins and membrane lipids. Thiyl radicals can react with GSH and ascorbate. On the other hand, the high constant rates of the reactions of thiyl radicals with chemical compounds present in high concentrations in cells reduce the effectiveness of antioxidants and the removal of the thiyl radicals, especially in lipid bilayers. It suggests that there is no dominant antioxidant specific to thiyl radicals. Moosmann and Hajieva dedicated a large part of their paper to thiyl radicals which arise from Cys (CysS^•^). This is an important aspect of the biochemistry of thiyl radicals because both the -SH group of free Cys as well as the -SH group of protein Cys can undergo one-electron oxidation, which in the latter case leads to the formation of protein thiyl radicals. Protein thiyl radicals can also be formed by homolysis of the disulfide bonds formed by protein cystine (Cys-Cys). It is well known that Cys is toxic to cells, and the causes of this phenomenon are not well understood [24]. Several mechanisms are considered here, among which the possibility of generation of toxic oxidized Cys derivatives is often discussed [25]. Moosmann and Hajieva put forward the hypothesis that this practically-irremovable reactivity of thiyl radicals, responsible for the toxicity of Cys, “forced” the evolutionary Cys depletion. In support, the authors remind, inter alia, the following facts: (a) compared to other amino acids, Cys is rare on the surface of proteins; (b) there is an inverse correlation between Cys usage and longevity in animals; (c) Cys is replaced by Cys persulfide or by selenocysteine in critical sites. These are just a few selected examples. We strongly encourage the readers to read the entirety of this very interesting review paper.

In conclusion, we believe that this Special Issue has indeed provided new information about this unusual element, sulfur, and its compounds. In nine papers, the authors have presented the most up-to-date information on the biological and pharmacological properties of essential sulfur compounds, as well as the methods of their determination in biological samples. Certainly, many of the problems raised here require further research because numerous questions and hypotheses have been put forward by the authors. Sulfur and its compounds still retain their secrets.
